# Analysis of the Basal Plane Dislocation Density and Thermomechanical Stress during 100 mm PVT Growth of 4H-SiC

**DOI:** 10.3390/ma12132207

**Published:** 2019-07-09

**Authors:** Johannes Steiner, Melissa Roder, Binh Duong Nguyen, Stefan Sandfeld, Andreas Danilewsky, Peter J. Wellmann

**Affiliations:** 1 Crystal Growth Lab, Materials Department 6 (i-meet), FAU Erlangen-Nuremberg, Martensstr. 7, D-91058 Erlangen, Germany; 2 Crystallography, Albert-Ludwigs-University Freiburg, Herrmann-Herder-Str. 5, D-79104 Freiburg i. Br., Germany; 3 Micromechanical Materials Modelling (MiMM), Institute of Mechanics and Fluid Dynamics, Technical University Bergakademie Freiberg (TUBAF), Lampadiusstr. 4, D-09599 Freiberg, Germany

**Keywords:** silicon carbide, physical vapor transport, basal plane dislocation, small angle grain boundary, thermal stress

## Abstract

Basal plane dislocations (BPDs) in 4H silicon carbide (SiC) crystals grown using the physical vapor transport (PVT) method are diminishing the performance of SiC-based power electronic devices such as pn-junction diodes or MOSFETs. Therefore, understanding the generation and movement of BPDs is crucial to grow SiC suitable for device manufacturing. In this paper, the impact of the cooldown step in PVT-growth on the defect distribution is investigated utilizing two similar SiC seeds and identical growth parameters except for a cooldown duration of 40 h and 70 h, respectively. The two resulting crystals were cut into wafers, which were characterized by birefringence imaging and KOH etching. The initial defect distribution of the seed wafer was characterized by synchrotron white beam X-ray topography (SWXRT) mapping. It was found that the BPD density increases with a prolonged cooldown time. Furthermore, small angle grain boundaries based on threading edge dislocation (TED) arrays, which are normally only inherited by the seed, were also generated in the case of the crystal cooled down in 70 h. The role of temperature gradients inside the crystal during growth and post-growth concerning the generation of shear stress is discussed and supported by numerical calculations.

## 1. Introduction

Bulk silicon carbide (SiC) grown using the physical vapor transport (PVT) growth method has been established as a new material for high performance power electronic devices [1,2]. To achieve high device performance it is essential to understand defect generation during crystal growth. Basal plane dislocations (BPDs) occurring in the (0001)-plane can degrade SiC pn-junction diodes after long term forward voltage operation [3] or increase the leakage current in blocking mode of SiC power MOSFETs [4] and JFETs [5]. Since BPDs lie perpendicular to the c-axis, i.e., the growth direction employed in PVT-growth, they cannot easily propagate into the grown crystal from the seed. Instead, they have to be generated either during the growth itself or after finishing the growth while the crystal is cooling down. Contrary to BPDs, threading edge dislocations (TEDs), threading screw dislocations (TSDs) and micropipes (MPs) will be inherited from the seed crystal due to their direction of propagation along the c-axis. In addition, the interaction between these types of defects will complicate the prediction of dislocation behavior during PVT growth [6,7]. 

Axial and radial temperature gradients inside the graphite crucible are necessary to promote growth and polytype stability. However, the variation of thermal gradients inside the grown crystal causes thermoelastic stress during the cooldown phase and can lead to the generation of dislocations such as BPDs [8]. Therefore, understanding the impact of the cooldown step on defect densities is important to grow low-defect SiC material. Numerical calculations have been performed by Gao et al. to investigate the effects of different cooling rates between 1 h and 10 h on BPD density distribution [9]. The conclusion was reached that faster cooling rates lead to decreased BPD densities. Residual compressive stress in the <11$\bar{2}$0> direction in the range of −763 to −490 MPa and tensile stress in the <1$\bar{1}$00> direction between 673 to 2953 MPa was reported by Xie et al. [10], indicating that a longer cooldown time after growth has an impact on BPD density due to annealing effects.

In this paper, two 100 mm 4H-SiC crystals are grown with short (40 h) and long (70 h) cooling steps, and their defect density is investigated using KOH-etching and birefringent imaging. Dislocation patterning is also discussed, including the <1$\bar{1}$00> and <11$\bar{2}$0> directions. Moreover, synchrotron white beam x-ray topography (SWXRT) measurements are conducted on wafers cut from the crystal of which the seeds are taken for crystals B and C to investigate the initial defect distribution. The wafers are cut 4° off-axis to reveal the BPDs puncturing the surface of the samples after KOH defect etching.

## 2. Materials and Methods

To investigate the effects of varying cooling rates on defect densities, two 100 mm 4H SiC-crystals named crystal B and crystal C were grown using the PVT-growth method, also known as the modified-Lely growth method. For the growth runs, two adjacent seeds B0 and C0 were taken from crystal A. The growth pressure for all three crystals A, B, and C was set to 5 mbar ambient pressure using Ar and N_2_, and the flow of nitrogen was set to 10% of the amount of the argon gas flow. The growth temperature at the seed was set to 2250 °C, verified by optical measurements using a pyrometer and numerical calculations. After the crystal growth was finished, an average cooling rate of 47.3 K/h (crystals A and B) and 24.6 K/h (crystal C) was achieved by ramping down the applied power to zero over a duration of 40 h and 70 h, respectively. An overview of the prepared samples is depicted in Table 1.

After growing, two wafers were cut near the growth interface and near the seed from each crystal, respectively, followed by a polishing step and characterization by birefringence imaging. Afterwards, they were etched in a 510 °C KOH-melt for 5–10 min to reveal the defect densities, in particular basal plane dislocations, and were subsequently examined utilizing differential interference contrast (DIC) microscopy. In addition, crystal A was analyzed using SWXRT (Karlsruhe Institute of Technology (KIT), Karlsruhe, Germany).

SWXRT measurements were done on wafers cut from crystal A in back-reflection geometry, the most suitable geometry for the determination of individual dislocations in (0001)-cut wafers [11,12]. A complete mapping of the 100 mm wafer was recorded in back-reflection geometry using the 0004 reflection with the aid of an indirect two-dimensional detector system, consisting of a scintillator crystal, an optical element, and a CCD camera pco 4000. The indicated beam size for the mapping of the sample was 5 mm × 7 mm. The distance between camera and sample amounted to about 100 mm.

## 3. Results and Discussion

### 3.1. SWXRT Analysis of Crystal A

A growing crystal will arrange itself according to the temperature field in which it grows. Therefore, previous to cooling down, the stress inside the crystal will be minimal and defects are for the most part inherited from the seed. However, once the cooling down phase begins, the temperature gradients inside the crystal will change as well, reaching zero when the crystal has completely cooled down to room temperature. This will cause additional stress within the crystal and, together with the initially high temperatures, promote plastic deformation by the generation and mobilization of defects such as BPDs and TEDs.

**Figure 1:**
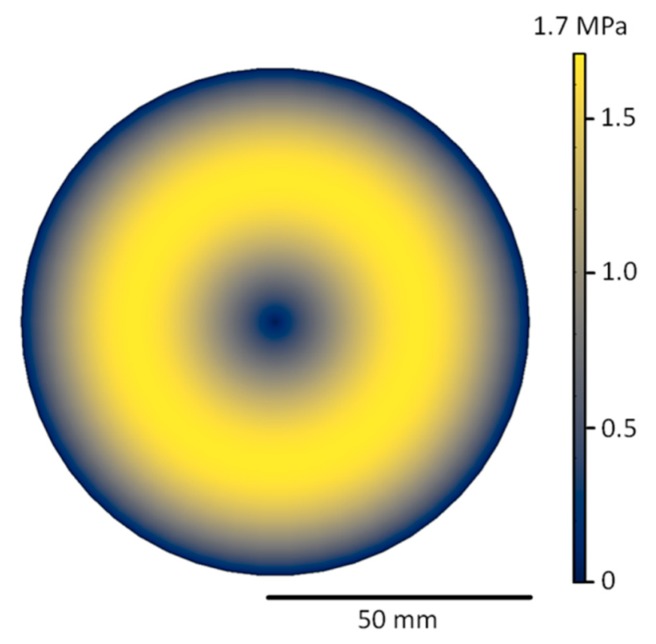
Calculated resolved shear stress distribution inherent to grown wafers at 2250 °C.

A numerical calculation was carried out to investigate the shear stress in the basal plane of the crystals grown in this paper. The aim of this calculation was to approximate the lateral stress present inside the grown crystal during the cooling down period subsequent to growth. It neglected stress due to the geometry of the hot zone or different thermal expansion coefficients of the graphite crucible or seed holder, focusing only on shear stress created by cooling down. The results depicted in Figure 1 show a torus-like distribution of shear stress with minima in the center and the side of the wafer. Selder et al. [13] analyzes the thermal stress distribution in growing SiC bulk single crystals utilizing a global simulation of heat and mass transfer. The authors conclude a critical resolved shear stress for BPD generation of 1 MPa, which is exceeded by the modeled shear stress with a maximum stress of 1.7 MPa. As a consequence, due to the changing temperature gradients, stress-induced BPD generation in the cooldown stage is expected. 

**Figure 2:**
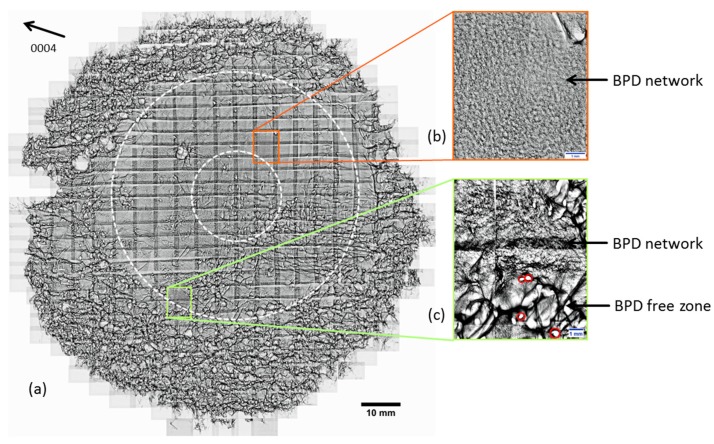
(**a**) Synchrotron white beam x-ray topography (SWXRT) mapping from a wafer cut from crystal A, recorded in back-reflection geometry in 0004 reflection; (**b**) section of the wafer exhibiting a network of dense basal plane dislocations (BPDs) distributed in a similar manner to shear stress presented in Figure 1, marked by dotted circles; (**c**) section of the wafer exhibiting a BPD network next to a zone defined by strain fields caused by domain boundaries.

To ensure a more in-depth investigation of the distribution of the different defect types from crystal A, SWXRT mappings recorded in back-reflection geometry were conducted. The results depicted in Figure 2 demonstrate the defects present inside the grown crystal A. Outside of the center of the wafer, a dense distribution of BPDs can be observed (illustrated in Figure 2b), while the density of TEDs, MPs and TSDs is comparatively low. This arrangement corresponds with the distribution of shear stress calculated in Figure 1. At the edge of the wafer, a high variety of defects, including TSDs connected by BPDs, can be observed in addition to MPs, whose strain fields are partially overlapping (depicted in Figure 2c). The BPD distribution predominantly located in the inner ring between the center and the edge of the wafer correlates with the calculated shear stress distribution indicated in Figure 1, while the patterning seen at the edge of the wafer mainly consists of TEDs forming domain boundaries arranged along the <1$\bar{1}$00> direction. Since TEDs lie parallel to the c-axis, and therefore in the growth direction, these defects will be inherited from the seed and propagate through the crystal during growth. Due to this, it can be assumed that crystals B and C, grown with seeds originating from this crystal, should exhibit a similar TED distribution.

### 3.2. Investigation of Tilt and Strain of Crystals B and C

**Figure 3:**
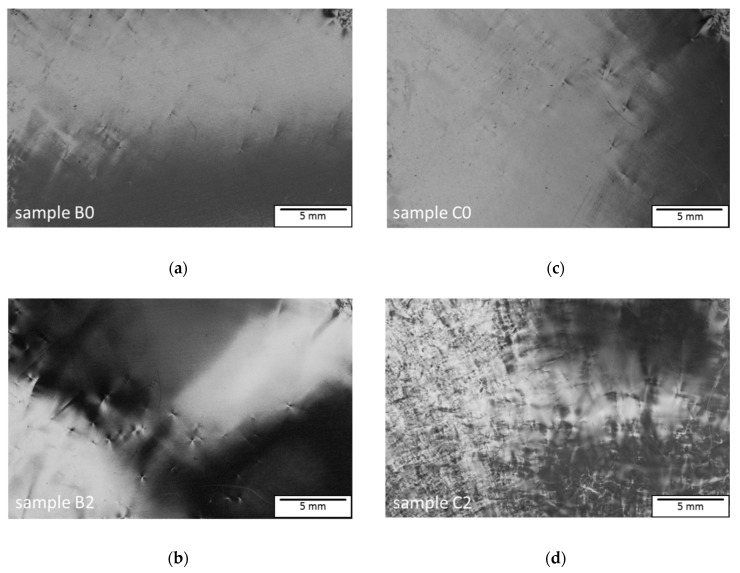
(**a**) Birefringence scan of sample B0, the seed utilized to grow crystal B; (**b**) birefringence scan of sample B2, cut near the crystal growth interface; (**c**) birefringence scan of sample C0, the seed utilized to grow crystal C; (**d**) birefringence scan of sample C2, cut near the crystal growth interface. The increased strain and tilt is clearly visible for sample C2, cooled within 70 h.

To investigate the impact of the different cooldown times on the strain and tilt in the grown crystals, birefringence imaging on the polished samples B2 and C2 was conducted and compared with the seeds B0 and C0, used to grow crystals B and C. In birefringence images, MPs are mostly visible due to their large burgers vector [14], but these scans also give a good approximation of the overall strain in the crystal lattice. In addition, the low angle grain boundaries consisting of TED structures seen in the SWXRT-mapping above should also be visible due to the tilt in the crystal lattice. Figure 3a,b illustrates a comparison between the seed B0 and the wafer B2, cut from the resulting crystal B using birefringence scans. The images were taken at the same position of the wafer, which can be verified by a characteristic defect cluster of MPs located in the upper right corner of both pictures. A similar density of strain caused by MPs and TSDs is visible for both wafers B0 and B2, indicating that the overall density of these types of defects has not changed significantly. This is expected, since the growth parameters for crystals A and B were the same, including a cooldown period of 40 h. The situation changes for samples C0 and C2, depicted in Figure 3c,d. The scans were again taken at the same position on the wafers. Sample C0 displays almost the same positions of defects as sample B0 because these two wafers were cut out of crystal A adjacent to each other. However, in sample C2, a high amount of strain and tilt caused by an increased amount of MPs, TSDs, and domain boundaries consisting of TED-arrays, is apparent. Due to the almost identical seeds B0 and C0 used in growing crystals B and C, the reason for the divergent strain and tilt of samples B2 and C2 has to lie in the different employed cooling durations of 40 h and 70 h, respectively. An explanation for this observation could be the fact that crystal C was held at a higher temperature for a longer time due to the prolonged cooldown phase. Consequently, crystal C will also be subjected to increased plastic deformation accomplished by the generation and motion of defects such as BPDs and TEDs. Since there are no domain boundaries detectable in seed C0, the strain seen in sample C2 had to occur by TED generation during the prolonged cooldown step.

### 3.3. BPD Density and Dislocation Patterning

**Figure 4:**
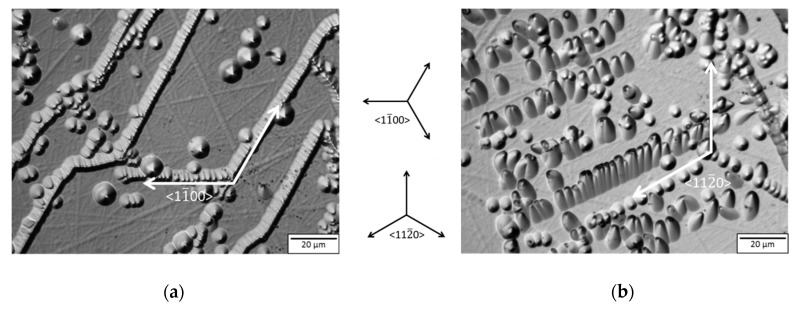

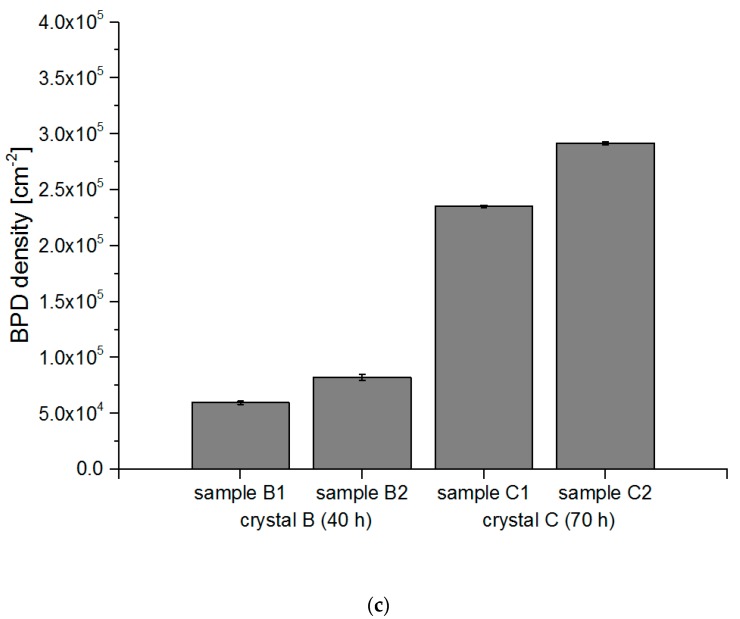
Differential interference contrast (DIC) optical microscope images of the (0001)-surface of 4° off-axis wafers etched with KOH; (**a**) Subdomain boundaries consisting of threading edge dislocations (TEDs) arranged along the <1$\bar{1}$00> direction, taken from sample C2 in the center area of the wafer; (**b**) array of basal plane dislocations (BPDs) lined up in the <11$\bar{2}$ 0> direction, taken from sample C2 in the middle between the center and the edge of the wafer. Both images are aligned according to the directions depicted in the middle; (**c**) defect density of BPDs derived from etched wafers, where samples B1 and C1 correspond to wafers cut next to the seed from crystal B and crystal C, respectively, while samples B2 and C2 correspond to wafers cut near the growth interface of crystal B and crystal C, respectively.

To verify the assumption of increased TED domain boundary generation in crystal C compared with crystal B, KOH defect-etching was conducted and the defect patterning investigated. Additionally, to study the development of BPD density in crystals B and C, the density was counted by statistical means on the etched wafers B1, B2, C1, and C2. The results are illustrated in Figure 4. Primarily, two types of pattern can be observed: Figure 4a shows an array of TEDs aligned in the <1$\bar{1}$00> direction. These domain boundaries have been described by Glass et al. [15]. They were partly inherited by the seed crystal, continued during growth, and were partly caused by thermoelastic stress [8]. Figure 4b, on the other hand, depicts arrays of BPDs piercing the surface due to the 4° off-axis angle in which the wafer was cut, aligned in the <11$\bar{2}$0> direction. The reason for these defects lies in plastic deformation during the cooling phase by slip and cross slip mechanisms [16] or conversion between TEDs and BPDs [17]. A proposal for the mechanism of the formation of low angle grain boundaries has been made by Dudley et al. [7], where quarter loops of BPDs and TEDs form between misoriented growth centers during growth and mobilize in the cooldown period driven by arising thermoelastic stress due to changing thermal gradients. While the cut wafers from crystal B exhibit a distribution of TED domain boundaries manifesting themselves mainly at the edge of the crystal in a manner similar to their seed, both wafers cut from crystal C with the slow cooling rate show TED-based domain boundaries stretching across the wafer contrary to the utilized seed. Since both crystals were grown with seeds adjacent to each other, and the only difference between crystal B and crystal C was the cooling rate, this indicates that these domain boundaries are not only determined by the seed and stress occurring during growth but are also impacted and generated due to the longer cooling time. This further emphasizes the importance of a cooling rate fast enough to prohibit dislocation generation at high temperatures where the plasticity of the grown crystal is high.

Figure 4c depicts the evolution of the BPD density of crystals B and C. It is apparent that crystal C, cooled with a rate of 24.6 K/h, exhibits a much higher density of BPDs compared with crystal B, cooled down with a rate of 47.3 K/h. Since BPDs cannot propagate easily in growth direction due to their movement being limited to the basal plane, they will not be inherited by the seed crystal. Furthermore, the fact that the same process parameters were used during both growth runs means that the reason for the deviating BPD density has to be found in the post-growth period. Due to the different cooling rates, the stress will relieve in different ways for these crystals. Gao et al. [9] investigated the influence of cooling rates between 1 h and 10 h on the BPD density numerically and concluded that fast cooling will inhibit the generation of BPDs and is therefore beneficial for defect reduction. Both the BPD density and the strain observed via KOH-etching and birefringent imaging confirm these conclusions, despite the fact that the cooling times with 40 h and 70 h were significantly longer than the 10 h discussed in the work of Gao et al.

## 4. Conclusions

Two 100 mm 4H-SiC crystals B and C were grown using the PVT-method, with adjacent seeds taken from crystal A. For crystals B and C, identical growth parameters were utilized except for a cooldown step of 40 h and 70 h, respectively. The seeds B0 and C0, used for growing crystals B and C, were taken from crystal A and exhibited similar defect distributions and densities. The impact of the prolonged cooldown step on defect density and patterning was investigated via birefringence imaging and KOH etching. Since all other growth parameters such as temperature gradients, hot zone geometry, and the fact that the seeds were basically the same, resulting differences can be attributed to the cooling down step. It was found that increasing the duration of the cooldown step not only significantly increased BPD density in the resulting crystal, as reported by Gao et al. [9], but also promoted a change in the distribution of small angle grain boundaries based on TEDs, also discussed by Dudley et al. [7]. Further investigations should be performed using a significant decrease of the cooldown phase to diminish the mobility and generation of defects post-growth, or by exploring the impact of variations in the amount of temperature gradients during growth on the resulting defect distribution.

## Figures and Tables

**Table 1:** Overview of the grown crystals and the prepared samples.

Prepared Samples	Source Crystal	Cooldown Rate [K/h]	Additional Notes
B0 ^1^	A	47.3	Seed for crystal B
C0 ^1^		47.3	Seed for crystal C
B1	B	47.3	Cut next to the seed
B2		47.3	Cut near the growth interface
C1	C	24.6	Cut next to the seed
C2		24.6	Cut near the growth interface

^1^ Sample B0 and C0 were cut out of crystal A adjacent to each other.

## References

[1] Wellmann P.J. (2018). Review of SiC crystal growth technology. Semicond. Sci. Technol..

[2] Wellmann P., Neubauer G., Fahlbusch L., Salamon M., Uhlmann N. (2015). Growth of SiC bulk crystals for application in power electronic devices—process design, 2D and 3D X-ray in situ visualization and advanced doping. Cryst. Res. Technol..

[3] Bergman P., Lendenmann H., Nilsson P.Å., Lindefelt U., Skytt P. (2001). Crystal Defects as source of anomalous forward voltage increase of 4H-SiC diodes. Mater. Sci. Forum.

[4] Agarwal A., Fatima H., Haney S., Ryu S.-H. (2007). A new degradation mechanism in high-voltage sic power MOSFETs. IEEE Electron Device Lett..

[5] Veliadis V., Hearne H., Stewart E.J., Snook M., Chang W., Caldwell J.D., Ha H.C., El-Hinnawy N., Borodulin P., Howell R.S. (2012). Degradation and full recovery in high-voltage implanted-gate SiC JFETs subjected to bipolar current stress. IEEE Electron Device Lett..

[6] Ohtani N., Katsuno M., Tsuge H., Fujimoto T., Nakabayashi M., Yashiro H., Sawamura M., Aigo T., Hoshino T. (2006). Behavior of basal plane dislocations in hexagonal silicon carbide single crystals grown by physical vapor transport. Jpn. J. Appl. Phys..

[7] Dudley M., Chen Y., Huang X.R., Ma R.H. (2008). Aspects of dislocation behavior in SiC. Mater. Sci. Forum.

[8] Schmitt E., Straubinger T., Rasp M., Weber A.D. (2006). Defect reduction in sublimation grown SiC bulk crystals. Superlattices Microstruct..

[9] Gao B., Kakimoto K. (2014). Three-dimensional modeling of basal plane dislocations in 4H-SiC single crystals grown by the physical vapor transport method. Cryst. Growth Des..

[10] Xie X., Hu X., Chen X., Liu F., Yang X., Xu X., Wang H., Li J., Yu P., Wang R. (2017). Characterization of the three-dimensional residual stress distribution in SiC bulk crystals by neutron diffraction. CrystEngComm.

[11] Dudley F., Huang W., Wang S., Powell J.A., Neudeck P., Fazi C. (1995). White-beam synchrotron topographic analysis of multi-polytype SiC device configurations. J. Phys. D Appl. Phys..

[12] Rack A., Weitkamp T., Trabelsi S.B., Modregger P., Cecilia A., dos Santos Rolo T., Rack T., Haas D., Simon R., Baumbach T. (2009). The micro-imaging station of the TopoTomo beamline at the ANKA synchrotron light source. Nucl. Instrum. Methods Phys. Res. B Beam Interact. Mater. Atoms.

[13] Selder M., Kadinski L., Durst F., Straubinger T., Wellmann P., Hofmann D. (2001). Numerical simulation of thermal stress formation during PVT-growth of SiC bulk crystals. Mater. Sci. Forum.

[14] Ouisse T., Chaussende D., Auvray L. (2009). Micropipe-induced birefringence in 6H silicon carbide. J. Appl. Crystallogr..

[15] Glass R.C., Kjellberg L.O., Tsvetkov V.F., Sundgren J.E., Janzén E. (1993). Structural macro-defects in 6H-SiC wafers. J. Cryst. Growth.

[16] Hull D., Bacon D.J. (2011). Introduction of Dislocations.

[17] Ha S., Mieszkowski P., Skowronski M., Rowland L.B. (2002). Dislocation conversion in 4H silicon carbide epitaxy. J. Cryst. Growth.

